# Better Together – Organizing a PAN-London IMG and Educators Conference

**DOI:** 10.1192/bjo.2024.282

**Published:** 2024-08-01

**Authors:** Harleen Kaur Birgi, Peter Carter

**Affiliations:** North East London NHS Foundation Trust, London, United Kingdom

## Abstract

**Aims:**

International medical graduates (IMG) are an important and integral part of the NHS workforce. The 2022 General medical council (GMC) Workforce Report showed that of the doctors who joined the workforce in 2021, half (50%) were IMGs and 39% UK graduates. This report also emphasized the need for better inclusion and support for IMGs in order to enhance future retention.

With this conference we aimed to empower IMGs and their educators with tools and knowledge to better recognize and help mitigate the challenges that IMGs endure whilst working in NHS. We discussed about the factors affecting IMG career progression, wellbeing, and ways to overcome them.

**Methods:**

It was a one-day conference targeted to the PAN-London cohort of IMGs and their educators of medical and surgical specialties. The programme included distinguished speakers from all branches of medical fraternity, the GMC and medical indemnity organisations. Five poster submissions were also selected to be presented on the day. The programme started with IMG consultants describing personal challenges and success stories with a focus on long-term NHS equality diversity inclusion plan. This was followed by an invigorating ‘Schwartz round’ wherein attendees were able to engage in open and reflective discussions of shared experiences in transition to the United Kingdom. The latter half of the day included workshops on mitigating differential attainment and medico-legal aspects of clinical practice. The conference was concluded by an informative discussion led by the head of GMC London.

**Results:**

The conference was well-attended with 94 attendees present on the day. The audience encompassed a varied set of professionals including medical education managers, directors of medical education, educational supervisors and IMG doctors of all grades and specialties across different London trusts. The feedback was overwhelmingly positive with all the respondents in agreement that the learnings from the conference were relevant to their professional needs. The qualitative response from the attendees in summary was that conferences of a similar agenda and focus should be organized in the future as well.

**Conclusion:**

Historically, there is clear evidence in literature that IMGs have lower success rates in both job and training progression, in comparison to British medical graduates. By organizing such conferences, the endeavor is to kick start a productive dialogue between IMGs and their educators, to target more favorable and successful overall outcomes, on a long-term basis. We hope that this initiative sets the building blocks for the way of the future.

